# HRD-MILN: Accurately estimate tumor homologous recombination deficiency status from targeted panel sequencing data

**DOI:** 10.3389/fgene.2022.990244

**Published:** 2022-09-28

**Authors:** Xuwen Wang, Ying Xu, Yinbin Zhang, Shenjie Wang, Xuanping Zhang, Xin Yi, Shuqun Zhang, Jiayin Wang

**Affiliations:** ^1^ School of Computer Science and Technology, Xi’an Jiaotong University, Xi’an, China; ^2^ Shaanxi Engineering Research Center of Medical and Health Big Data, Xi’an Jiaotong University, Xi’an, China; ^3^ Department of Oncology, The Second Affiliated Hospital of Xi’an Jiaotong University, Xi’an, China; ^4^ Geneplus-Beijing Institute, Beijing, China

**Keywords:** homologous recombination deficiency, targeted panel sequencing, multi-instance learning model, cancer genomics, sequencing data analysis

## Abstract

Homologous recombination deficiency (HRD) is a critical feature guiding drug and treatment selection, mainly for ovarian and breast cancers. As it cannot be directly observed, HRD status is estimated on a small set of genomic instability features from sequencing data. The existing methods often perform poorly when handling targeted panel sequencing data; however, the targeted panel is the most popular sequencing strategy in clinical practices. Thus, we proposed HRD-MILN to overcome the computational challenges from targeted panel sequencing. HRD-MILN incorporated a multi-instance learning framework to discover as many loss of heterozygosity (LOH) associated with HRD status to cluster as possible. Then the HRD score is obtained based on the association between the LOHs and the cluster in the sample to be estimated, and finally, the HRD status is estimated based on the score.

In comparison experiments on targeted panel sequencing data, the Precision of HRD-MILN could achieve 87%, significantly improved from 63% reported by the existing methods, where the highest margin of improvement reached 14%. It also presented advantages on whole exome sequencing data. Based on our best knowledge, HRD-MILN is the first practical tool for estimating HRD status from targeted panel sequencing data and could benefit clinical applications.

## 1 Introduction

Homologous recombination repair deficiency (HRD) usually refers to a state of Homologous Recombination Repair (HRR) dysfunction at the cellular level. HRD is a more stable molecular marker of malignancy (Moore et al., 2019), whose positive status is often found in various malignant tumors including ovarian, breast, and pancreatic ductal cancers (Pellegrino et al., 2020). Clinical studies have shown that cancer patients with HRD-positive status present highly sensitive to platinum-based chemotherapy and poly (ADP-ribose) polymerase (PARP) inhibitors (Konstantinopoulos et al., 2015; Telli et al., 2016). Thus, estimating HRD status in breast/ovarian cancer patients can expand the benefit population and improve prognosis (González-Martín et al., 2019; Ray-Coquard et al., 2019; Fasching et al., 2021). The development of PARP inhibitors as high-grade serous carcinoma of the ovary, fallopian tube, or peritoneum (HGSC) therapy resulted from the observation that BRCA mutations significantly increased the *in vitro* susceptibility of cancer cells to PARP inhibition (Bryant et al., 2005; Farmer et al., 2005).

Unfortunately, estimating HRD status is a complicated computational problem. The initial idea detects the related germline variations (Hoppe et al., 2018) or somatic mutations (GSM) on BRCA1/2 genes (Bell et al., 2011). But later studies reported a lot of negative examples (Mirza et al., 2016; Coleman et al., 2017; Pujade-Lauraine et al., 2017). It is suggested that more markers should be considered in HRD estimation. Powered by genome sequencing, current methods estimating HRD status are all NGS data-based (Sztupinszki et al., 2018). There are state-of-the-art methods for whole-exome sequencing (WES) or whole-genome sequencing (WGS) data. At present, the popular clinical sequencing assays for HRD have four categories: HRR-related gene mutation assays (Ledermann et al., 2016; Sherill-Rofe et al., 2019), Genomic Instability Score (GIS) (Alexandrov et al., 2015), mutation signature (Alexandrov et al., 2013; Alexandrov et al., 2020), and HRD functional assays. The clinical validity of HRD functional assays has not been well confirmed (Miller et al., 2020), as each type of method has its limitations, especially when handling targeted panel sequencing data. In the Background section, we discuss the computational issues on targeted panel sequencing data in detail.

Here, we provide HRD-MILN, a novel machine learning-based approach for estimating HRD status. It accurately and efficiently captures the genomic features of LOH from targeted panel sequencing data. Since it is hard to model the unclear/non-significant associations between a LOH mutation on the genomic level and the HRD status on the patient level, we use a supervised learning information imprecise multi-instance learning (MIL) framework to solve the critical computational issue. Comparison experiments on real sequencing data validate the MIL model. Based on our best knowledge, HRD-MILN is the first practical tool for estimating HRD status from panel sequencing data and could benefit clinical applications.

## 2 Background

The initial biomarker for HRD is GSM on BRCA1/2 genes (Bell et al., 2011; Kanchi et al., 2014). It is soon reported insufficient because HRR involves dozens of known genes, and abnormalities in these genes may also contribute to the HRD phenotype (Lee and Kopetz, 2022). There is no clear evidence that HRD can also arise through GSM or methylation of a broader set of HRR-related genes or other as-yet-undefined mechanisms (Radhakrishnan et al., 2014). Furthermore, clinical studies showed that it as a biomarker for predicting PARPi or platinum responses in HGSC patients cannot currently be established (Swisher et al., 2009; Swisher et al., 2017; Bernards et al., 2018). Some scholars emphasize mutational signature (MS) as a novel biomarker for judging HRD (Davies et al., 2017; O’Kane et al., 2017). Another opinion suggests the somatic copy number variations (SCNVs) imply genomic scars (Miller et al., 2020), e.g., telomeric allelic imbalance (TAI) (Birkbak et al., 2012) and large-scale state transition (LST) (Popova et al., 2012). From the ARIEL studies of rucaparib, LOH status can be a biomarker of PARPi response (Mirza et al., 2016). Thus, HRD biomarkers have three categories: 1) GSM based, 2) copy number variation based, and 3) LOH based. As LOH is composed of mutations and copy number variations, LOH is considered the most potential efficient biomarker (Abkevich et al., 2012).

The existing methods for estimating HRD status are developed based on the different HRD biomarkers or combinations. Some approaches are based on GSM in HRR-related genes (including BRCA1/2). Although GSM in BRCA1/2 significantly increased the *in vitro* Sensitivity of cancer cells to PARP inhibition (Bryant et al., 2005; Farmer et al., 2005), it is not sufficient. Furthermore, GSM in other HRR-related genes is associated with distinct sensitivities to PARPi (Marshall et al., 2019). Some approaches are based on the MS. This strategy analyzes MS mainly relies on mutational features, transcriptional strand bias, genomic distribution, and association analysis with genomic features to cluster and transform each type of mutation into a visual pattern. This type of approach has achieved good results in estimating cancers with HRD. However, it needs as much genome-wide information as possible is likely to offer greater specificity and Sensitivity (Miller et al., 2020), e.g., MutationalPatterns (Blokzijl et al., 2018) and YAPSA (Hübschmann et al., 2021). So they might work for WES or WGS (Goldfeder et al., 2016) but not for targeted panel sequencing. Moreover, this strategy lacks clinical evidence to support the efficacy prediction of PARP inhibitors, and its application is objectively limited by using paraffin-embedded samples for clinical testing. Some other approaches are based on genomic scar. There are two commercially available assays, the tumor BRCA mutation assays with an unweighted sum of GIS or the assessment of the sub-chromosomal LOH portion of the genome (Telli et al., 2016). For GIS, BRCA mutation-positive or GIS score ≥42 can be considered HRD-positive (Telli et al., 2016). The LOH test’s predefined cut-off of 14% or more defines LOH-high. It is deemed to be positive for HRD (Bell et al., 2011). The utility of LOH or GIS showed good clinical validity in their ability to determine the BRCAwt subgroups that benefited more from PARPi in the relapse platinum-sensitive setting (Miller et al., 2020). However, the accuracy of existing strategies of LOH or GIS is based primarily on the accuracy of the SCNV assay by the number of genomic scars for patient tumor samples. The current SCNV detection tools cannot accurately detect genomic scars. These false-positive genomic scars can misclassify the sample as false-positive for HRD.

Moreover, there were discrepant results in the HRD scores in different races, cancer species, and lifestyles or living conditions (Pellegrino et al., 2020). Thus, estimating the HRD status by a uniform threshold is problematic, an unweighted sum of GIS. Most importantly, such methods generally require high DNA loading, sequencing data volume, GSM covering the HRR signaling pathway, etc. The genomic distribution of target regions is often sparse and uneven on targeted panel sequencing data (Li et al., 2012; Talevich et al., 2016). Therefore, TAI and LST cannot be obtained, which may lead to low GIS on the panel data, thus misclassifying the sample to be tested as negative for HRD.

Some machine learning (ML) based approaches use HRD biomarkers to build ML models for estimating HRD status (Watkins et al., 2014; Chao et al., 2018; Nguyen et al., 2020). For example, HRDetect (Davies et al., 2017) used a lasso logistic regression model to identify six distinguishing MS predictive of BRCA1/BRCA2 deficiency (Gulhan et al., 2019). Using a machine learning approach instead of a single metric threshold approach has more significant advantages. It can effectively solve the accuracy problems and lack of generalization of the traditional HRD score calculation method. However, these methods have two disadvantages. 1) The premise of using supervised learning algorithms is that we have access to the labels of the training instances. However, we do not know the intrinsic connection between HRD biomarkers and HRD. These markers are only a manifestation of HRD, i.e., we cannot get the label of genomic scars to determine HRD (Watkins et al., 2014; Telli et al., 2016). 2) Due to the limitation of targeted panel sequencing, only LOH can be obtained, and obtaining other genomic scars information is difficult. Therefore, most methods are developed for WGS or WES but not targeted panel sequencing data.

## 3 Materials and methods

We proposed a new method to estimate HRD status based on a multiple instance learning framework. It is not reasonable that the existing ML model often adopts an aggressive strategy to obtain the training data (Davies et al., 2017; Miller et al., 2020): For an HRD-positive patient, assign all LOH (or LST, TAI) calls of this patient’s positive labels from a medical view. Meanwhile, the false-positive genomic scars can also affect the accuracy of this strategy (Miller et al., 2020). The latest research now suggests that there must be an association between LOHs and assessment of HRD status (Miller et al., 2020), which means it is certain that the presence of one or more LOHs makes the sample positive for HRD, but precisely what that association is not yet clear. Our research has two main steps to estimate HRD status based on a multiple instance learning framework. One step is identifying the potential association pattern between the LOHs and HRD status during training. Another step is to calculate the HRD score based on the association between the LOHs and the clusters in the sample to be estimated. This way, we can estimate the HRD status without giving the LOH label.

### 3.1 Identifying the potential association pattern between the LOHs and HRD status

In our research, we can’t get the label of LOH for estimating the HRD status. Therefore, we adopt the MIL (Maron and Lozano-P\'rez, 1997), which does not need category labels of instances, and the training package has category labels. Here, we set every sample as a package and each LOH status in every package as an instance. The core idea of the multi-instance learning method was that if one instance in the package were close enough to the calculated target concept point, it would be considered positive. However, due to the difference and complexity of the individual samples, the complexity and diversity of LOH, or the inaccuracy of the detection results, we modified multi-instance learning by proposing k target concept points (LOHs cluster) for detecting HRD. The input of HRD-MILN is a LOH file (TSV format), which is the result file of FACETS (Shen and Seshan, 2016) detection LOH. The output of our model is the HRD score, which is the prediction of the HRD status by HRD-MILN for a cancer sample. We collected 56-panel capture and 44 whole-exome sequencing samples to develop our model. All these samples are labeled with HRD status.

#### 3.1.1 Features selection for LOH

The initial feature dimension of LOH is 14. However, redundant features may affect the performance of the models. Therefore, we also performed feature selection for HRD-MILN. Due to MIL being different from MI, we used two steps (Ablation studying and Calculating the importance of each feature) for feature selection for LOH. First, the number of maximum practical features is calculated by Ablation studying. Then, the valuable features of LOH are selected by Calculating the feature’s importance.

#### 3.1.2 Ablation studying

To the candidate the adequate number of features, we adopt the strategy of ablation experiment based on MILBoost (Viola et al., 2007). MILBoost is a feature selection method for MIL, which focuses on feature selection through the boosting framework. In our ablation experiments, we first fix random seeds and then observe and analyze the change in model performance as the number of features decreases. In each number-of-features experiment, i.e., when the number of features is fixed, we randomly select different features and perform 50 experiments, taking the mean value as a result. For each number of features, we served 100 experiments and finally took the mean value as the final result. We use the default parameters of MILBoost. Our experimental results show that the number of practical features of LOH is 9 (Supplemental Figure S1), so the default number of LOH features in HRD-MILN is 9. Of course, the reader can candidate the most effective number of LOHs according to their data type.

#### 3.1.3 Calculating the importance of the feature

**FIGURE 1:**
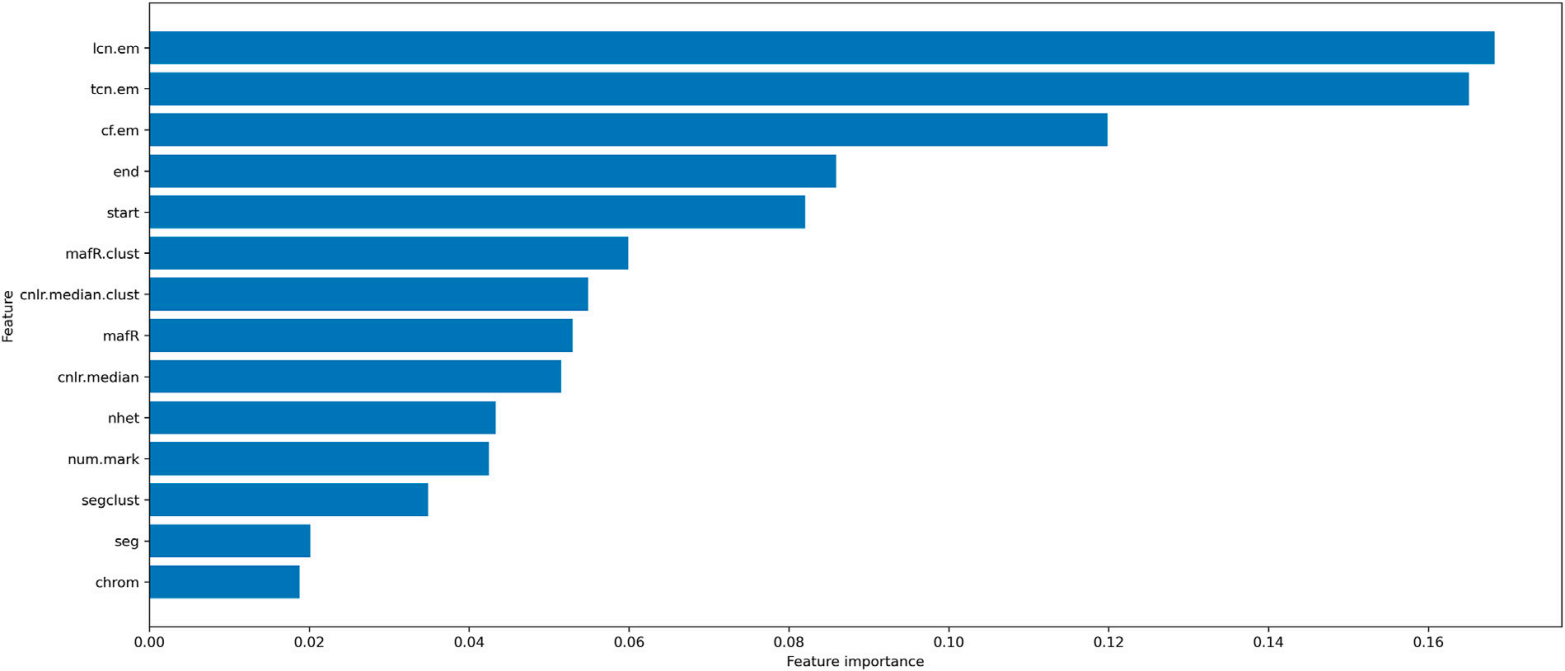
The importance of LOH features.

The initial features of LOH are (chrom, num. mark, nhet, cnlr. median, mafR, segcl, cnlr. median.clust, mafR.clust, start, end, cf. em, tcn. em, lcn. em). We used MILBoost to calculate each LOH feature’s importance for developing the model (Figure 1). Combining the adequate number of features and Figure 1, we finally selected the following nine features, including (nhet, cnlr. median, mafR, mafR.clust, start, end, cf. em, tcn. em, and lcn. em) (Table 1), as well as the adequate number of features and the feature importance of LOH. On the other hand, as a machine learning framework, although we have prior knowledge of some features, these features of LOH may not directly imply HRD-positive susceptibility. Here, we analyzed these features according to the training data. We believe that more biological or medical research will explain the potential susceptibility in the future. Next, the min-max normalization is used on the feature attributes. We scaled the attribute data with a significant difference, which would fall into a small interval to improve the algorithm’s convergence speed and detection accuracy.

**TABLE 1:** The specific meaning of every feature attribution.

Feature	Specific meaning
chrom	The No. of chromosome
seg	ID number in this segment started from 1
num.mark	Detection intervals contained in this segment
nhet	Heterozygous SNP included in this segment
cnlr.median	The median of the copy number log ratio in this segment
segclust	This segment cluster was based on tcn and icn
cnlr.median.clust	The median of the copy number log ratio in this cluster
start	The start position
end	The end position
mafR	The summary statistic of log odd-ratio as described
mafR.clust	The summary statistic of log odd-ratio as described in this cluster
cf.em	The em value of the cell content in this segment
tcn.em	The em value of the total copy number in this segment
lcn.em	The em value of the less copy number in this segment

##### 3.1.4 The LOHs cluster for estimating HRD status

**FIGURE 2:**
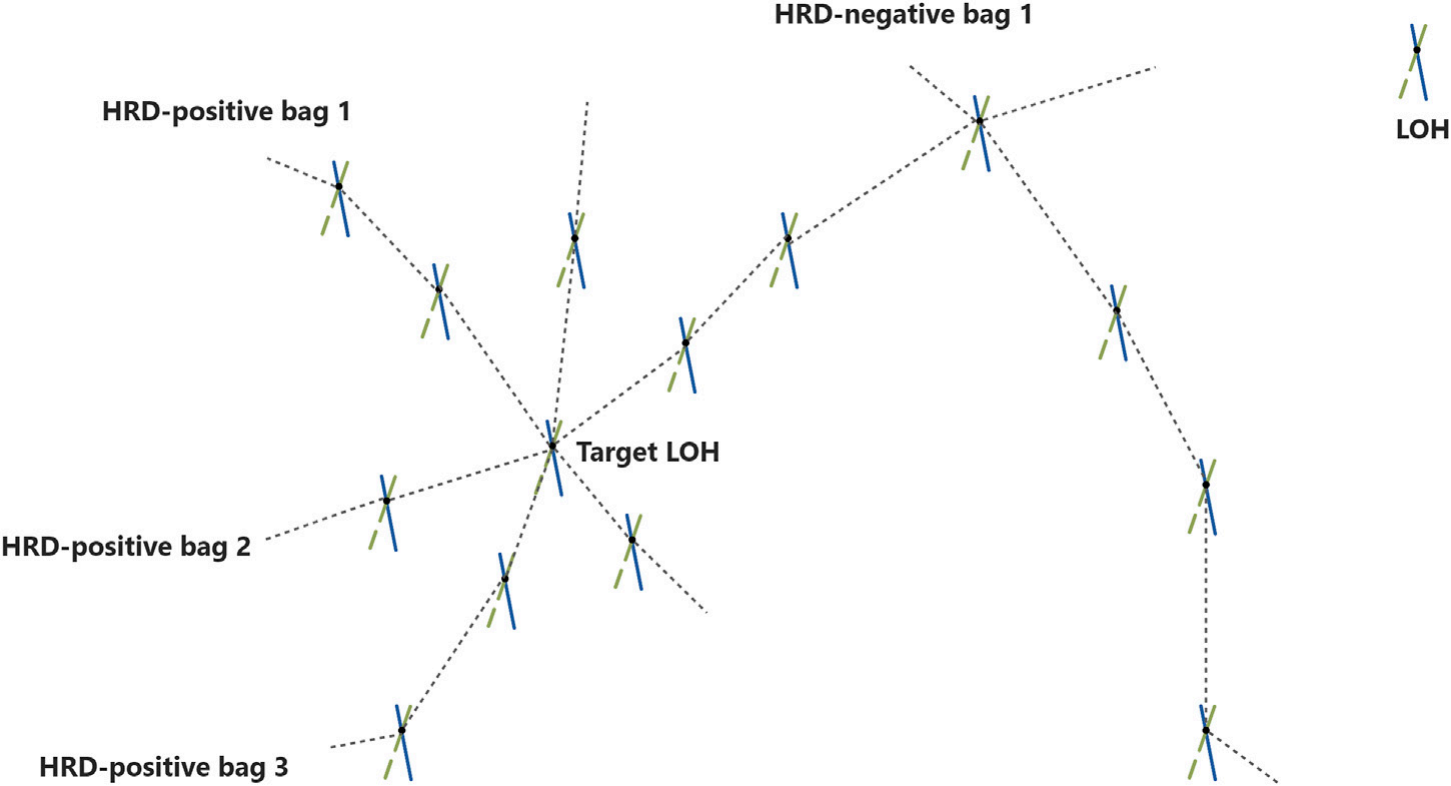
The schematic diagram of the target point. Showed in the figure, an instance was regarded as a point, and one package had multiple instances. The trajectories of these instances were treated as a manifold. For example, in point A, this intersection should satisfy that every positive package passed through this point, and no negative package passed through this point. So, point A may be a target point.

As shown in Figure 2, the LOH instance was regarded as a point, and one sample package had multiple LOH instances. The trajectories of these LOHs were treated as a manifold. For example, in point A, this intersection should satisfy every positive package passed through this point, and no negative sample package passed through it (It may be a target LOH). LOH instances and sample packages were subjected to a particular probability distribution. The diversity density (DD) (Maron and Lozano-P\'rez, 1997) function value of a LOH instance was this point’s probability value, satisfying the potential positive or negative sample package distribution. One LOH instance had a DD value to find the max DD value as the target concept LOH. Then we used this target concept LOH as a reference to calculate the distance between every LOH and this LOH and then determine the HRD status of this sample through whether the minimum distance was within the threshold. Based on the complexity of our research content, we propose a strategy of multiple target concept LOHs and gather them into a cluster (named the LOHs cluster). Finally, the LOHs in the samples to be estimated are clustered with the LOHs cluster, and this process can filter the false-positive LOHs and LOHs that are not related to HRD status.

##### 3.1.5 Identifying the association between the LOHs cluster and HRD status

The influence of every characteristic on the label could be modeled in the DD algorithm by associating an unknown factor. The target concept LOH, which means hot spot LOH, consisted of two values the ideal attribute value and the scale value. $T=\left\{t_{1},t_{2},\cdots ,t_{K}\right\}$ represented the target concept LOHs, $t_{k}=\left\{t_{k1},t_{k2},\cdots ,t_{km}\right\}$ , *t*_*nd*_ represented *d* ^*th*^ feature of *t*_*k*_, *m* is the dimension of the feature, and $label\left(B_{i}|T\right)$ represented the prediction of *B*_*i*_ with *T*. *B*_*i+*_ represented the *i* ^*th*^ HRD positive sample, *B*_*ij+*_ represented the *j* ^*th*^ LOH instance in the *i* ^*th*^ HRD positive sample. *B*_*ijd+*_ described the *d*_*th*_ feature of LOH in the *j* ^*th*^ LOH instance of the *i* ^*th*^ HRD positive sample. The same as the *B*_*i-*_, *B*_*ij-*_, *B*_*ijd-*_.

$$\mathrm{Pr}\left(t=t_{k}|B_{ij}\right)=\exp\left\{-{\overset{m}{\underset{d=1}{\sum }}\left(s_{d}\left(B_{ijd}-t_{kd}\right)\right)}^{2}\right\}$$ (1)

Pr is denoted as the probability of the LOH becoming a potential target concept point, defined as the distance between the LOH and the target concept point. The similarity between the conformation and the ideal shape increased, and the bending strength decreased exponentially. Eq. 1 was used to determine if *T* is a real target concept of LOH. Since some of the features may be uncorrelated or need a higher weight, thus we used the weighted Euclidean distance (Ungerboeck, 2006) to measure the distance between LOHs, and *s*_*d*_ represented the weight of the *d* ^*th*^ feature. The initial default value of *s*_*d*_ is 1. Readers can set it according to their own data.

### 3.2 Efficiently solving the model by modifying the EMDD algorithm

A single target concept LOH was used in the traditional experiment using the multi-instance learning method (Zhang and Goldman, 2001) algorithm (e.g., EMDD) to detect HRD status. However, the prediction of the category of the package would bed unsatisfactory. EMDD is a very typical and widely used multi-instance learning method. Thus, we choose EMDD as the research object and improve it to verify the effectiveness of our proposed multiple target concept points in detecting HRD. Our model processes a standard TSV file containing LOH and outputs a regular TSV file. To ensure that the relationship between K target LOHs is low, we used the weighted Euclidean distance to measure the distance among points. So, our strategy is suitable to solve the hot spots and randomness problems of LOH for HRD detection. The main steps are as follows.

E-step: We selected several initial LOH instances that were most likely to be labeled, different from the traditional EMDD algorithm. Then we used the current hypothetical target t to estimate the most probable label LOH in each training package (here means sample), and these LOH instances represented their respective packages. The *Pro* was used as the threshold of reliable candidate target LOH instances.

$$label\left(B_{i}|T\right)=\frac{\overset{K}{\underset{i=k}{\sum }}p_{k}^{*}}{K},p_{k}^{*}>Pro,p_{k}^{*}\in \mathrm{Pr}\left(B_{ij}|t=t_{k}\right)$$ (2)

$$NNLDD\left(T,D\right)=\frac{\overset{K}{\underset{k=1}{\sum }}\overset{n}{\underset{i=1}{\sum }}\left(-logPr\left(l_{i}|t_{k}^{\prime },B_{i}\right)\right)}{K}$$ (3)

$$t^{\prime }=\left\{t_{1}^{\prime },t_{2}^{\prime },\cdots ,t_{k}^{\prime }\right\},\prod \mathrm{Pr}\left(l_{i}|t_{k}^{\prime }\right)>Pro,k\in K$$ (4)

Here, *Label(Bi|T)* or *l*_*i*_ represented the candidate label of *Bi*. *p*_*i*_ * meant the possibility of candidate label of *Bi* with the target point *i*. *K* represented the number of target points. *NNLDD* represented DD values of *K* target points. *t'* represented the new concept point.

M-step: According to the above Eq. 2 Eq. 3 Eq. 4, we used the gradient ascent method to obtain the *K* new concept points *t'* for these training examples. Then we used the *t'* to replace the *t* in the E-step. Repeat E-step and M-step until the difference between the adjacent *t* values converges.

### 3.3 Calculating the HRD score to estimate HRD status

By the above steps, we can get the LOHs in the sample to be estimated similar to the LOHs cluster. Then, according to these LOHs, calculate the HRD score. Briefly as below: Bi represented the *i* ^*th*^ sample to be expected, *B*_*ij*_ represented the *j* ^*th*^ LOH instance in the *i* ^*th*^ sample to be predicted. *B*_*ijk*_ described the *k*_*th*_ feature of LOH in the *j* ^*th*^ LOH instance of the *i* ^*th*^ sample. Next, we can calculate the probability of it being LOH instance positive (the HRD score) by Eq. 6. Then we can determine whether the LOH to be tested is a LOH instance positive by Eq. 5, Eq. 7. As long as there was a LOH instance positive in a sample, the sample was labeled positive HRD status, otherwise negative HRD status.

$$f_{HRD-MILN}\left(B_{i}\right)=\left\{ \begin{array}{c} +1,\exists f\left(B_{ij}\right)=+1\\ -1,\exists f\left(B_{ij}\right)=-1 \end{array}\right.,\left(1\leq i\leq n,1\leq j\leq n\right)$$ (5)

$$Pro\left(B_{ij}\right)=\frac{\overset{K}{\underset{k=1}{\sum }}\exp\left\{-{\overset{n}{\underset{d=1}{\sum }}\left(s_{d}\left(B_{ijd}-t_{kd}\right)\right)}^{2}\right\}}{K}$$ (6)

$$f\left(B_{ij}\right)=\left\{ \begin{array}{c} =+1,Pro\left(B_{ij}\right)\geq NNLDD_{thre}^{*}\\ =-1,Pro\left(B_{ij}\right)\leq NNLDD_{thre}^{*} \end{array}\right.$$ (7)

Here, *f*_*HRD-MILN*_(*Bi*) means the prediction of the HRD status of HRD-MILN. *NNLDD*_*thre*_ * means the sample’s probability threshold is HRD positive, trained by E-step and M-step.

### 3.4 Data collection and bioinformatics pipeline

#### 3.4.1 Targeted panel sequencing samples

We had panel capture sequencing data of 56 cancer samples with known HRD status, including 28 HRD positive and 28 HRD negative samples. And these samples were provided by Gene+, Inc. We worked with the BAM files, which were obtained from https://db.cngb.org/.

#### 3.4.2 Whole-exome sequencing samples

##### 3.4.2.1 Study design and patients

In the meantime, subjects recruited for this study included a subset of clinically diagnosed breast cancer (25 individuals) and ovarian cancer (19 individuals) patients in the Department of Oncology, the Second Affiliated Hospital of Xi’an Jiaotong University (Approval No. 2022038). The institutional review and privacy boards reviewed this trial at all sites. All patients provided written informed consent.

##### 3.4.2.2 WES and LOH analysis

This cohort’s available tumor tissues from 44 patients underwent whole-exome sequencing (WES). Genomic DNA was obtained from formalin-fixed, paraffin-embedded (FFPE), or aspirated biopsy tumor specimens and blood samples QIAamp DNA FFPE Tissue Kit and DNeasy Blood Tissue Kit (Qiagen, United States), respectively, and analyzed using the dsDNA HS detection kit (ThermoFisher Scientific, United States). All samples were sequenced on an Illumina Hiseq4000 instrument using the 150 PE protocol (Illumina, United States). The quality control of FASTQ files is dealt with by Trimmomaticc (Bolger et al., 2014). Paired-end reads were then mapped to the human reference genome (hg19) using BWA-MEM (v.0.7.15) (Li and Durbin, 2009). Duplicate reads were marked by the MarkDuplicates tool in Picard. GATK3 was used to process the resulting BAM files to correct mapping and base quality score recalibration (Van der Auwera et al., 2013). We used ContEst (Broad Institute, contamination rate <0.02) to estimate Cross-sample (Cibulskis et al., 2011). We used Mutect (Cibulskis et al., 2013) and Scalpel (Fang et al., 2016) to call Somatic Single Nucleotide Variant and insertion/deletions. We used Snp-pileup (Shen and Seshan, 2016) to generate a CSV file containing SNV information on each chromosome from each dataset’s Bam file (BamN and BamT). Then, we used Facets (Shen and Seshan, 2016) to generate a *_cncf. TSV file containing copy number variations from the results of the Snp-pileup.

## 4 Results

To evaluate the performance of HRD-MILN, we conduct experiments on real datasets, which contain panel capture sequencing data of 56 cancer samples with known HRD status (28 HRD positive samples and 28 HRD negative samples), and a subset of WES samples of clinically diagnosed breast cancer (25 individuals) and ovarian cancer (19 individuals) patients. First, we did multiple experiments to demonstrate that multiple target LOHs affect the algorithm’s accuracy in detecting HRD. We also did several experiments to compare the proposed method and the original algorithm (EMDD). Finally, we also did several experiments to compare the performance of detecting HRD between HRD-MILN and the existing algorithm (SigMA) (Gulhan et al., 2019). In addition, the performance of the above methods is quantified by Precision, Sensitivity, and f1-score, where *precision = TP/(TP + FP), sensitivity = TP/(TP + FN), and f1-score* is the harmonic mean between the *Precision* and *Sensitivity. TP* is the number of true positive HRD samples, FP denotes the number of false positive HRD samples, and *FN* represents the number of false negative HRD samples. Their default parameters are used to compare our method with existing ones fairly.

### 4.1 Application of HRD-MILN to targeted panel sequencing samples

Prediction of HRD status was a binary classification problem (Ledermann et al., 2016). Due to the small samples, the experiment used the 10-fold cross-validation method and Nested cross-validation. To select an appropriate target concept point and the appropriate Pro threshold, 500 sets of experiments were done. Through multiple sets of pre-experiments, we set the default Pro threshold of candidate target concept points to 0.9 (Table.2). Meanwhile, to verify the necessity of the HRD-MILN method to predict the HRD status based on the panel sequencing data, a total of 500 sets of experiments were also done. The variance of the 10-fold cross-validation is 0.00084. Nested cross-validation is very suitable for small-sample machine learning modeling. Varma et al. (Varma and Simon, 2006) show in their paper that the test set error obtained using nested cross-validation is almost the correct error. Comparing the scores of nested cross-validation with the regular procedure (Supplemental Figure S2) shows that the average difference is 0.000522 with std. dev. of 0.000920, it is again demonstrated that our proposed method is still valid in the case of small samples. The specific accuracy of the different targets model is shown in Table.3. From Table.3, it could be seen that the candidate target was 3, the average scores of Precision, Sensitivity, and f-score were all of the best, and the accuracy of each group was not fluctuate much. Note that the difference between our model and EMDD is that the number of target points is different (*k* vs. 1), and the number of the target point of EMDD is 1. According to the particularity of the HRD samples, it was necessary to improve the EMDD algorithm and propose constructing a multiple target concept point to assist decision-making in improving the accuracy of HRD detection. On the other hand, due to the particularity and complexity of HRD, introducing too many targets may introduce time complexity and background noise, which would affect the final experimental results.

**TABLE 2:** Results under different Pro thresholds for selecting target points. Abbreviations: *h*: hour. *Pro*: the threshold of reliable candidate target points.

*Pro*	0.85	0.90	0.95	0.97
Average Targets	4	3	2	1
Average Time	2 h	1 h	0.5 h	0.3 h

**TABLE 3:** Model accuracy in different targets on the panel sequencing data.

Groups	Average Targets	Precision	Sensitivity	F1-score
Group 1	1	0.25	0.5	0.33
	2	0.74	0.73	0.73
	3	0.89	0.88	0.88
	4	0.80	0.79	0.79
Group 2	1	0.76	0.54	0.63
	2	0.80	0.77	0.78
	3	0.87	0.86	0.86
	4	0.84	0.80	0.82
Group 3	1	0.76	0.54	0.63
	2	0.75	0.73	0.74
	3	0.85	0.85	0.85
	4	0.76	0.73	0.74
Group 4	1	0.52	1	0.68
	2	0.88	0.88	0.88
	3	0.91	0.91	0.91
	4	0.92	0.91	0.91
Group 5	1	0.82	0.71	0.76
	2	0.78	0.70	0.73
	3	0.79	0.75	0.77
	4	0.77	0.68	0.72
Group 6	1	0.76	0.54	0.63
	2	0.84	0.80	0.82
	3	0.90	0.89	0.89
	4	0.89	0.88	0.88
Group 7	1	0.76	0.54	0.63
	2	0.75	0.70	0.72
	3	0.89	0.89	0.89
	4	0.88	0.84	0.86
Group 8	1	0.81	0.80	0.80
	2	0.85	0.82	0.83
	3	0.88	0.87	0.87
	4	0.81	0.80	0.80
Group 9	1	0.90	0.88	0.89
	2	0.83	0.73	0.78
	3	0.91	0.90	0.90
	4	0.86	0.86	0.86
Group 10	1	0.83	0.75	0.79
	2	0.79	0.62	0.69
	3	0.86	0.85	0.85
	4	0.74	0.68	0.71

**FIGURE 3:**
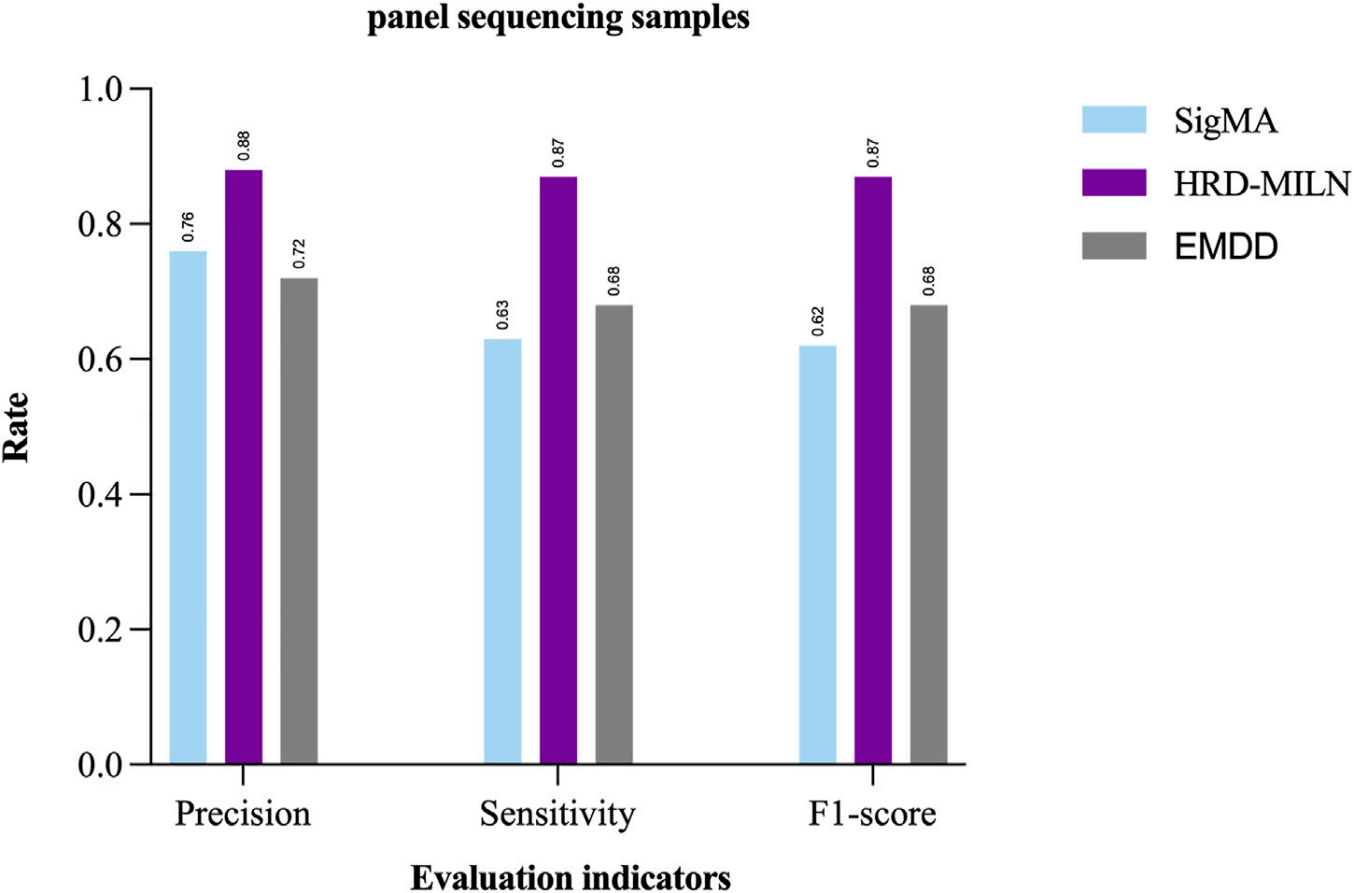
The performances of SigMA, EMDD, and HRD-MILN in panel sequencing samples. HRD-MILN had the highest scores (precision 0.88, sensitivity 0.87, F1-score 0.87) than SigMA and EMDD on each Evaluation indicator.

Due to the difference and complexity of the individual samples, the complexity and diversity of LOH, or the inaccuracy of the detection results, it is hard for the EMDD algorithm (a single target concept in general) to detect HRD. Therefore, according to the characteristics of our study content, the EM was improved to help us better determine the HRD status of tumor samples more accurately. We compared the results tested by the two methods (Table.4; Figure 3). The improved HRD-MILN (the average Precision is 0.88, Sensitivity is 0.87, F1-score is 0.87) is significantly better than EMDD (the average Precision is 0.72, Sensitivity is 0.68, F1-score is 0.70) in detecting HRD. To verify the effectiveness and necessity of the HRD-MILN method for panel sequencing data, we compared HRD-MILN with SigMA, the best model for detecting HRD based on panel sequencing data (Figure 3). HRD-MILN had higher scores (with a precision of 0.88, sensitivity 0.87, F1-score 0.87) than SigMA (with precision 0.76, sensitivity 0.63, F1-score 0.62) on each Evaluation indicators. This also proved the validity, accuracy, and necessity of HRD-MILN.

**TABLE 4:** HRD-MILN vs. EMDD. We compared various aspects of HRD detection performance of HRD-MILN and EMDD on the panel sequencing data. Abbreviations: HRD: Homologous recombination deficiency.

	Methods	Precision	Sensitivity	F1-score
Group 1	EMDD	0.25	0.5	0.33
	HRD-MILN	0.89	0.88	0.88
Group 2	EMDD	0.76	0.54	0.63
	HRD-MILN	0.87	0.86	0.86
Group 3	EMDD	0.76	0.54	0.63
	HRD-MILN	0.85	0.85	0.85
Group 4	EMDD	0.52	1	0.68
	HRD-MILN	0.91	0.91	0.91
Group 5	EMDD	0.82	0.71	0.76
	HRD-MILN	0.79	0.75	0.74
Group 6	EMDD	0.76	0.54	0.63
	HRD-MILN	0.90	0.89	0.89
Group 7	EMDD	0.76	0.54	0.63
	HRD-MILN	0.89	0.89	0.89
Group 8	EMDD	0.81	0.80	0.80
	HRD-MILN	0.88	0.87	0.87
Group 9	EMDD	0.90	0.88	0.88
	HRD-MILN	0.91	0.90	0.90
Group 10	EMDD	0.83	0.75	0.79
	HRD-MILN	0.86	0.85	0.85

**FIGURE 4:**
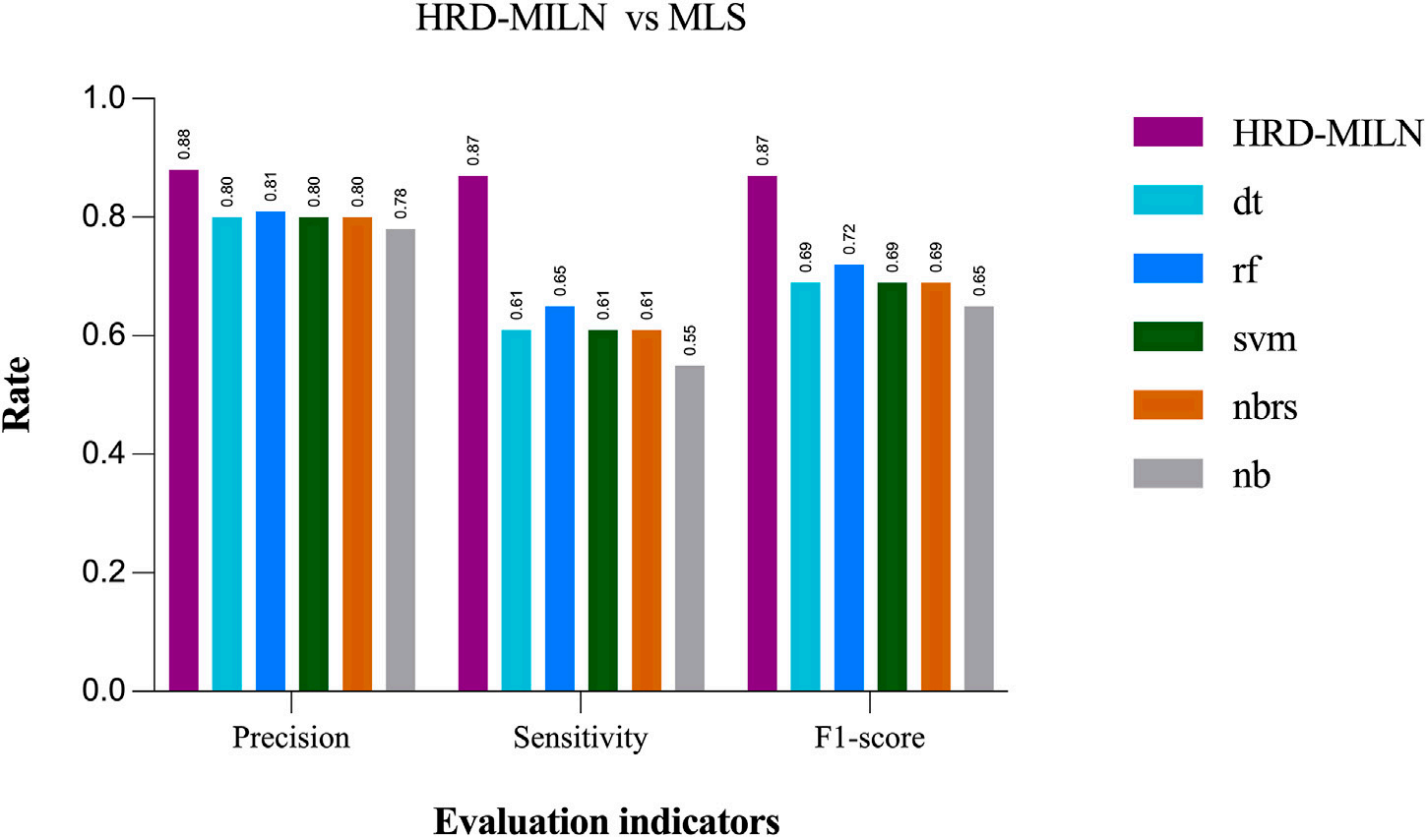
The performances of MILS and HRD-MILN in panel sequencing samples. HRD-MILN had the highest scores (precision 0.88, sensitivity 0.87, F1-score 0.87) than MILS on each Evaluation indicator. Abbreviations: MLS: machine learnings, dt: DecisionTree. rf: Random Forest. svm: Support Vector Machine. nbrs: KNeighbors. nb: GaussianNB.

At the same time, we also compared HRD-MILN with ML for detecting HRD based on panel sequencing data (Figure 4). HRD-MILN had higher scores (with a Precision of 0.88, the Sensitivity of 0.87, F1-score 0.87) than machine learnings (MLS) (the best ML scores are precision 0.81, sensitivity 0.65, F1-score 0.72) on each Evaluation indicators. This also proves that ML is not suitable for detecting HRD. The MLS compared in our experiments are DecisionTree (dt) (Quinlan, 1986), Random Forest (rf) (Breiman, 2001), Support Vector Machine (svm) (Cho and Prabhu, 2002), KNeighbors (nbrs) (Blough et al., 2006), GaussianNB (nb) (De Moraes and Machado, 2008).

### 4.2 Application of HRD-MILN to whole-exome sequencing samples

To evaluate the effectiveness of our proposed method, we also chose to validate it on the WES sequencing samples. These samples included a subset of patients diagnosed with breast cancer (25 individuals) and ovarian cancer (19 individuals) in the Department of Oncology, the Second Affiliated Hospital of Xi’an Jiaotong University. For a fair comparison, we compared HRD-MILN with SigMA and EMDD by using the default parameters for each model.

**FIGURE 5:**
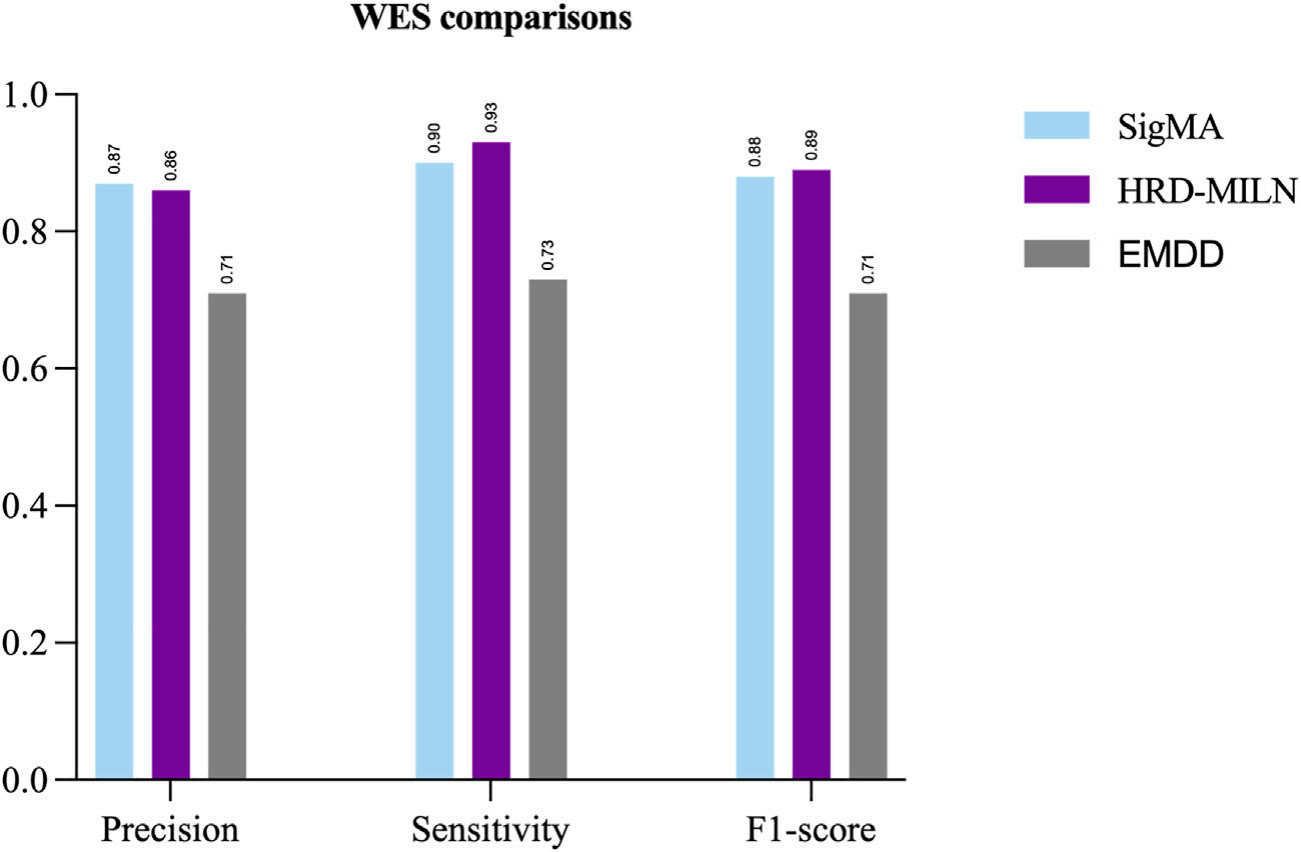
The performances of SigMA, EMDD, and HRD-MILN in whole-exome sequencing samples. HRD-MILN had the highest scores (precision 0.86, sensitivity 0.93, F1-score 0.89) than SigMA and EMDD on each Evaluation indicator.

**FIGURE 6:**
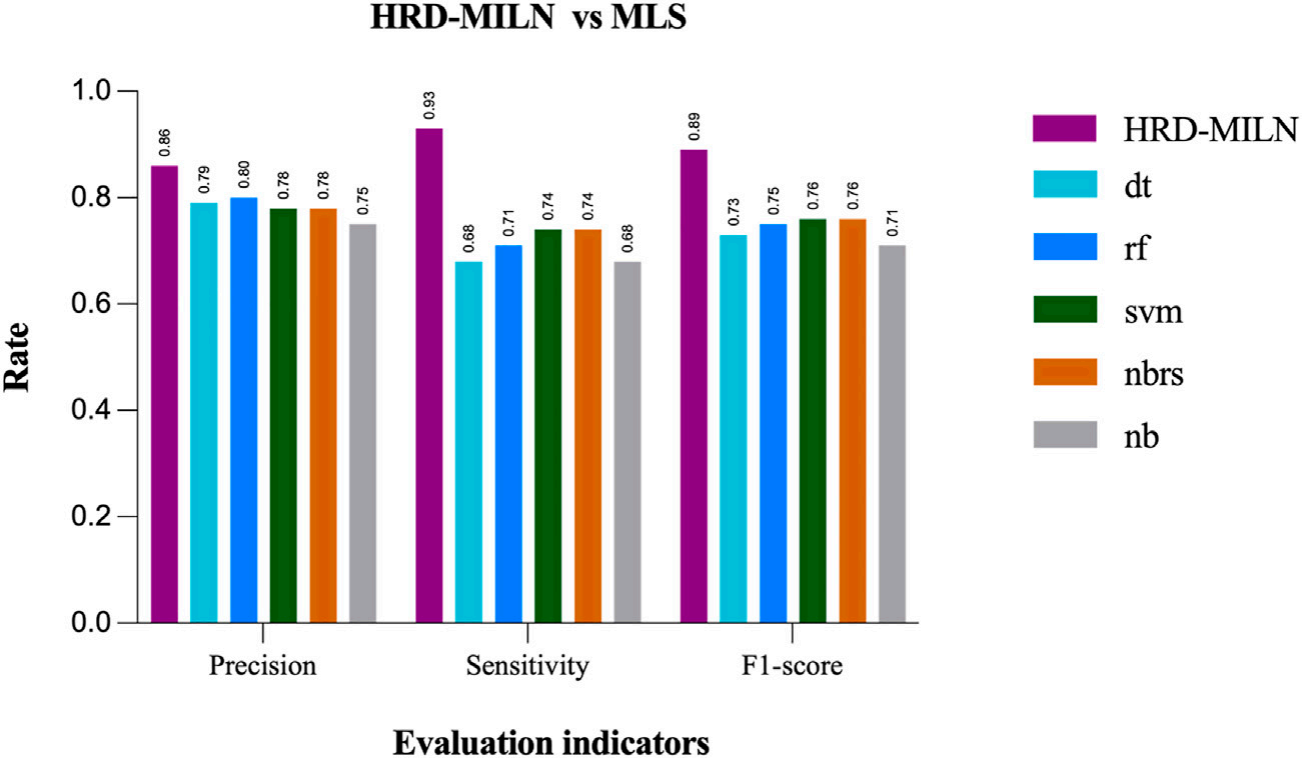
The performances of MILS and HRD-MILN in whole-exome sequencing samples. HRD-MILN had the highest scores (precision 0.86, sensitivity 0.93, F1-score 0.89) than MILS on each Evaluation indicator. Abbreviations: MLS: machine learnings, dt: DecisionTree. rf: Random Forest. svm: Support Vector Machine. nbrs: KNeighbors. nb: GaussianNB.

First, we tested the effect of different numbers of target points on the performance of HRD-MILN. Through multiple sets of pre-experiments, we set the default Pro threshold of candidate target concept points to 0.9 (Table.2). Meanwhile, to verify the necessity of the HRD-MILN method to predict the HRD status based on the WES sequencing data by bootstrapping (sampling 500 random sets of real samples with replacement). Here we show only four sets of experimental results. The specific accuracy of the different targets model is shown in Table.5. From Table.5, it could be seen that the candidate target was 3. The average scores of Precision, Sensitivity, and f1-score were all of the best (0.86, 0.93, 0.89) compared with (0.73, 0.81, 0.77) for 4 targets, the second-best performer. The accuracy of each group did not fluctuate much. This result was the same as the panel sequencing data. It again justifies our improvement strategy of using multiple target concept points to detect the status of HRD for cancer samples. Next, we compared the HRD-MILN with SigMA and EMDD on the WES sequencing samples. The results (Figure 5) show that HRD-MILN achieved the best average Precision of 0.86, Sensitivity of 0.93, and F1-score of 0.89 on the WES sequencing. SigMA is the second-best performer (0.87, 0.90, 0.88). From Figure 3, HRD-MILN was significantly better than SigMA and EMDD. Each method had relatively balanced scores on each Evaluation metric. The performance of EMDD is worse than HRD-MILN. This demonstrates that the improved approach is more effective for detecting HRD. Meanwhile, we compared HRD-MILN with ML for detecting HRD based on whole-exome sequencing data (Figure 6). HRD-MILN had higher scores than MLS (the best ML scores are precision 0.78, sensitivity 0.74, F1-score 0.76) on each Evaluation indicators. This also proves that ML is not suitable for detecting HRD on WES.

**TABLE 5:** Model accuracy in different targets on the WES data.

Groups	Average Targets	Precision	Sensitivity	F1-score
Group 1	1	0.76	0.54	0.63
	2	0.62	0.62	0.62
	3	0.85	0.92	0.88
	4	0.63	0.57	0.6
Group 2	1	0.67	1	0.8
	2	0.7	0.73	0.71
	3	0.85	0.93	0.89
	4	0.73	0.86	0.79
Group 3	1	0.73	0.66	0.69
	2	0.67	0.73	0.7
	3	0.85	0.93	0.89
	4	0.72	0.96	0.82
Group 4	1	0.69	0.71	0.7
	2	0.81	0.87	0.84
	3	0.88	0.93	0.9
	4	0.84	0.86	0.85

## 5 Discussion and conclusion

Accurately estimating HRD status is a challenging computational problem in cancer genomics and is also a bottleneck preventing from identifying potential benefits to patients. The mutational events on the HRR pathway and genomic scars (LOH, LST, TAI) suggest HRD estimation biomarkers. The existing ML model often adopts an aggressive strategy to obtain the training data: For an HRD-positive patient, assign all LOH (or LST, TAI) calls of this patient’s positive labels. This strategy is not reasonable from a medical view. There are no significant associations between one LOH (or LST, TAI) at the genomic level and the HRD status at the patient level. Literature suggests that those biomarkers, many of which may be similar to passenger somatic mutations, may randomly occur on genomes. But another opinion considers those functional biomarkers identical because they are induced as genomic scars. Thus, the multi-instance learning framework seems the best solution at present to model the complicated associations/similarities. In this study, we incorporated multi-instance learning in a novel way. For the training instance, a LOH has complex genomic features. It also implies an individual difference. The complexity of LOH leads to the design of multiple target concept points. Thus, we selected more than one target concept point, which improved the accuracy and Sensitivity as expected. Thus HRD-MILN cloud solves the key computational issue that it is hard to model the unclear/non-significant associations between a LOH mutation on the genomic level and the HRD status on the patient level. And by establishing the intrinsic associations among HRD biomarkers and HRD status, HRD-MILN is much less sensitive to false positive mutation calls (e.g., LOHs) than the existing methods.

Targeted panel sequencing is the most popular sequencing strategy in clinical practices, not only because of the high cost-performance ratio but also due to governmental policies. It will keep the top cancer sequencing service providers over the coming years. Thus, it is meaningful to develop this tool for targeted panel sequencing data and hopefully could benefit cancer patients. In addition, the proposed method could also be used on WES and WGS data. The experiments demonstrated that HRD-MILN consistently outperforms the existing methods on different sequencing data, which should be helpful for widespread clinical applications.

In the future, we will pursue two experimental aims. First, HRD clinical samples are challenging to collect. Although the number of HRD samples in this study is ‘big data’ compared to clinical studies, it is limited compared to model development. Therefore, we will continue to collect more HRD samples to verify the validity of the proposed method. Second, it would be worthwhile to investigate whether other clinical computing problems with HRD could benefit from HRD-MILN.

## Data Availability

The original contributions presented in the study are included in the article/Supplementary Material, further inquiries can be directed to the corresponding authors.
