# Supplementary figures and images for: HRD-MILN: Accurately estimate tumor homologous recombination deficiency status from targeted panel sequencing data
Source: Front Genet. 2022 Sep 28;13:990244. doi: 10.3389/fgene.2022.990244 (PMC9554509; doi:10.3389/fgene.2022.990244)

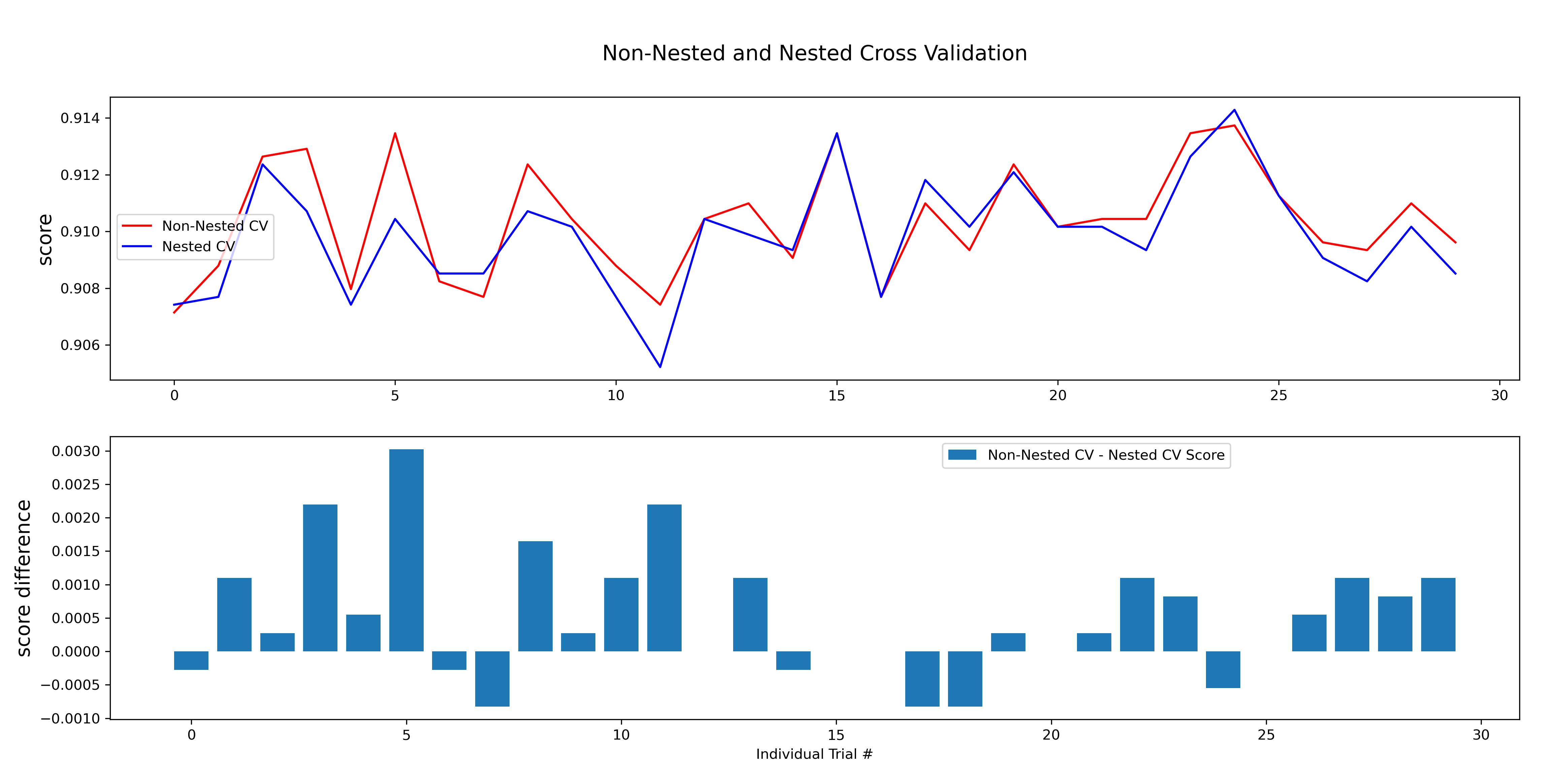

Supplement: Supplementary file 1 [file Image2.TIF]

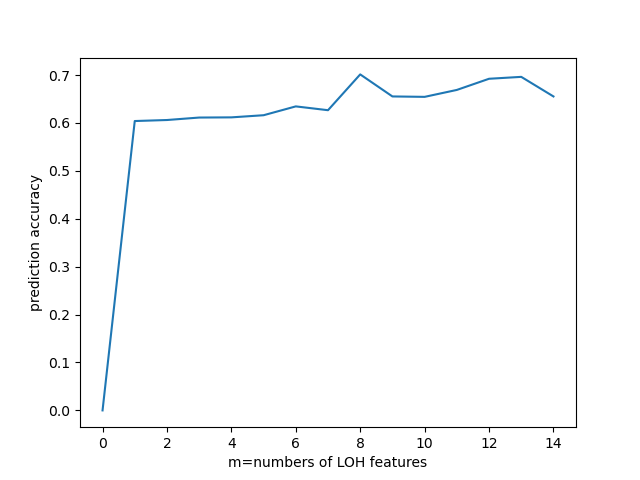

Supplement: Supplementary file 2 [file Image1.TIF]
